# Reflections on 50 Years of Cystic Fibrosis Newborn Screening Experience with Critical Perspectives, Assessment of Current Status, and Predictions for Future Improvements

**DOI:** 10.3390/ijns11040088

**Published:** 2025-09-30

**Authors:** Philip M. Farrell

**Affiliations:** Departments of Pediatrics and Population Health Sciences, University of Wisconsin School of Medicine and Public Health, 600 Highland Avenue, Madison, WI 53792, USA; pmfarrell@wisc.edu; Tel.: +1-608-345-2308

**Keywords:** cystic fibrosis, newborn screening, meconium, cystic fibrosis transmembrane regulator gene, immunoreactive trypsinogen, nutrition, lung disease, cystic fibrosis transmembrane conductance regulator-related metabolic syndrome (CRMS), cystic fibrosis screen positive, inconclusive diagnosis (CFSPID)

## Abstract

The morbidity/mortality risks of cystic fibrosis (CF) with a delayed diagnosis have made newborn screening (NBS) attractive for the past 50 years. Initial efforts focused on meconium analyses, but these proved unsatisfactory. After dried blood spot specimens became valuable for NBS applied to other genetic disorders and immunoassay methods became routine, the discovery of immunoreactive trypsinogen (IRT) led to numerous CF NBS programs around the world. Excellent laboratorians led the way, but CF clinicians rightly questioned the benefit–risk relationship and unanswered questions about IRT. These issues were resolved by the combination of a positive randomized clinical trial and the discovery of the cystic fibrosis transmembrane conductance regulator gene (*CFTR*) and its principal pathogenic variant, F508del. Recommendations for universal screening and then the proliferation of IRT/DNA screening programs followed. But more knowledge has brought more complexity, including an enigmatic, distracting condition known as cystic fibrosis transmembrane conductance regulator-related metabolic syndrome (CRMS) or cystic fibrosis screen positive, inconclusive diagnosis (CFSPID). Recently, with the recognition that CF is not a “white person’s disease,” and that over 1000 *CFTR* pathogenic variants occur, attention has turned to achieving equity and timeliness for all babies. Continuous quality improvement has characterized the past decade, as greatly expanded *CFTR* panels in the DNA tier through next-generation sequencing offer promise and raise the prospect of a primary genetic screening test.

## 1. Introduction

The rationale for the early diagnosis of cystic fibrosis (CF) has been appealing since comprehensive, partially effective treatment programs emerged [1,2]. CF newborn screening (NBS) became compelling when Shwachman et al. [3] published data revealing a dramatic difference in survival when children were diagnosed under 3 months of age presymptomatically or when mildly affected compared to those requiring hospitalizations [4]. Thus, Shwachman [4] became an early advocate for NBS and convincingly described his passion to me in January 1975 when we attended a conference together [5]. I vividly recall a frank, inspiring discussion during the break after the session entitled “Prenatal and Neonatal Screening.” My CF mentor, Paul di Sant’Agnese [6], had just made a startling statement in the session that the sweat test would never be adequate because of a “flaw,” namely the requirement that an astute primary care physician must first order the test. Dr. Shwachman agreed and told me that the only hope was NBS and that “Phil, as a neonatologist and CF Center Director in Wisconsin you should be the one to advance the field.”

During the subsequent 50 years of my involvement in CF NBS, its advantages and numerous challenges have become clear. It has also become well established that, CF NBS must function as a synergistic hybrid of public health and healthcare operations to be fully effective and expedite the integration of the multiple steps. Now, I find it informative to reflect critically on the journey towards an optimized diagnosis through NBS. It is a story about international collaborations, controversies, and never-ending quality improvement (QI).

Despite the many accomplishments and countless QI efforts, glaring shortcomings are evident when one conducts a critical review of the current status, particularly in comparison to NBS for other genetic conditions. But a natural tendency has prevailed to evaluate CF NBS and its outcomes compared to the prescreening era [6] rather than in the context of NBS per se—the benchmark preferred by my 2007–2008 sabbatical mentor, Harry Hannon, director of the newborn screening branch of the Centers for Disease Control and Prevention (CDC) [7]. I believe that he was correct and more rigorous standards of timeliness, equity, and optimized care remain desirable goals, particularly to achieve better outcomes for all children with CF. Although prescreening versus post-screening comparisons were useful in the early years of IRT-based NBS, i.e., from around 2000 to 2010, treatments were evolving then, especially with the development of CFTR modulator therapy.

### Purpose of This Review and Commentary

Although Travert et al. [8] published an excellent review of the “early years,” their description of the “trajectory” 5 years ago focused on the laboratory tests, reflecting the authors’ area of special expertise. This commentary was presented at the 2025 European Cystic Fibrosis Society (ECFS) conference and critically describes the evolution of CF NBS in a broader historical context, with attention to incremental advances built on new knowledge and emerging replacement technologies, the associated outcomes, and the obvious shortcomings. Hopefully, it will provide a more current view and identify future needs. Accordingly, my intention is to summarize the CF NBS history critically and comprehensively from my personal/professional perspectives, describe an assessment of the status, and conclude with predictions for future improvements to address the current needs. The laboratory and clinical perspectives shared in this article will hopefully be as instructive to others as to me when my review and literature analysis for the 4 June 2025 ECFS Neonatal Screening Working Group meeting informed five provocative predictions described at the end of this commentary. Although I have had the privilege to advise eight European countries along with two Canadian provinces, Chile, and India, most of my experience has been in the USA, so with apologies, I will concentrate on American progress.

## 2. CF Newborn Screening Explored with Meconium Analyses

After the ill-fated “Kiss Your Baby” campaign initiated in 1973, meconium protein analyses emerged as the first promising screening strategy. NBS for CF was attempted during the 1970s in several programs, as reviewed by Stephan et al. [9], using the technique of meconium analysis for elevated protein concentrations. A dye-impregnated paper strip marketed by the Boehringer-Mannheim Corporation as the BMC-meconium kit was typically used. The screening test was performed at the crib side or in a nearby lab, dipping the strip into the initial meconium excreted, followed by paper chromatography that could reveal through a gray-blue color increased concentrations of predominantly albumin in babies with pancreatic insufficiency, but probably not pancreatic sufficiency [9,10,11,12,13]. This test was applied in the region of Veneto [11] during 1974 (where screening with improved methods has continued for 51 years) and in several European cities, plus in Boston, by Shwachman [12] and Wisconsin [10]. My use of this test, beginning in 1977, quickly revealed its shortcomings, particularly since the interpretation of the color change was so subjective and both false positive and false negative results were frequent, as reported by Bruns et al. [10]. In addition, the potential for mass processing was obviously limited by the labor-intensive aspect, in contrast to the use of dried blood spot (DBS) specimens originally developed by Guthrie et al. [14] for phenylketonuria screening and applied effectively for the much more common challenge of congenital hypothyroidism [15]. From a broader perspective, however, the meconium-based screening era was both advantageous and disadvantageous. The advocates of CF NBS like Shwachman and Mastella were even more motivated and encouraged, despite the shortcomings of the test, while the opposition stiffened because of the lack of test validity and the potential harm of causing psychosocial stress.

## 3. CF NBS Gains Traction with IRT

The first of three pivotal, enabling advances was reported in 1979 from research in New Zealand by Crossley et al. [16]. This development occurred because NBS labs had become skilled at performing immunoassays for thyroxin in congenital hypothyroidism screening [15], a radioimmunoassay for “immunoreactive trypsin” had been developed by Elias et al. [17] and reported in 1977, and the DBS cards were routinely stored. Jeanette Crossley, in collaboration with the Auckland CF Center director, Robert Elliott, completed a brilliant retrospective evaluation of immunoreactive trypsinogen (IRT) concentrations on DBS specimens stored from children who had been diagnosed with CF. This first laboratorian–clinician partnership was a perfect collaboration between a skilled, practical NBS leader and a distinguished CF specialist with a previous interest in screening with the use of timed aquagenic wrinkling. Their research was successful beyond belief because of its rapid, catalytic impact. Importantly, their observations on IRT were quickly confirmed by other leaders of NBS labs with immunoassay expertise, particularly Georges Travert in Normandy, France, and Anthony and Mary Heeley in Peterborough, England [8]. Concurrently, in New South Wales, Australia, Bridget Wilcken applied her broad expertise in NBS to generate supportive data [18] and, with some political encouragement, began screening neonates routinely during 1981 with an IRT/IRT program applied to a relatively a large birth population that totaled 1.2 million initial IRT tests when her landmark paper was published [19]. During the 1980s, the appealing DBS-based test led to similar programs elsewhere, including Colorado, as NBS labs drove the initial progress in CF NBS [20]. The results consistently showed that the value of IRT/IRT far exceeded the BMC-meconium test, which quickly became obsolete.

In retrospect, this was an exciting period for many laboratorians as IRT/IRT programs proliferated, but to others, especially some CF clinical leaders, it seemed alarming that such rapid, widespread implementation was occurring without enough critical research on the outcomes. It is noteworthy, however, that under the leadership of Keith Hammond and Frank Accurso, another invaluable partnership, Colorado transformed the use of IRT/IRT into a tool for pioneering clinical research linked to their NIH-funded Clinical Research Center with studies that clarified nutritional issues [21].

Despite the excitement, the “early years” [8] failed to clarify a variety of issues about IRT as an analyte, including the inconsistencies and variations in the biomarker results, the potential benefits of early diagnosis, and the risks/harms of CF NBS. A troubling issue that remains today is the best strategy for setting IRT cutoff values on both the initial DBS specimen and the follow-up sample. Moreover, the timing of the follow-up (recall) specimen and what threshold level to use for the further follow-up decision, which is still an inadequately studied issue, continue to be challenges.

There were two IRT kits available in the 1980s—one from Sorin Biomedica (Italy) and the other from Hoechst Behring (Germany) that was used by Crossley et al. [16]. For practical purposes, the initial IRT cutoff was typically set higher than optimal for a better specificity, causing up to 20% false negative results and much confusion, even as it is currently used. An important issue that emerged relates to the seasonal variations in the IRT results due to its temperature liability, but there are also variations that occur from kit to kit. It was hoped that when PerkinElmer began to supply more consistent kits with better analytical procedures, this problem of unpredictable variations might be resolved. However, the challenge continues and affects the goal of achieving equity. For example, without the floating IRT cutoff method, babies born in the summer have a lower probability of being detected with fixed IRT cutoff values compared to winter births.

Thus, a Task Force convened by the U.S. Cystic Fibrosis Foundation (CFF) identified in their 1983 report [22] several disconcerting issues related to unanswered questions about IRT, such as the analyte’s reliability and validity, its age-related declines, the hazards of a repeat/recall specimen (still a concern), and a need to standardize the assay. The Task Force recommended more research on IRT to address these and other questions, especially the biomarker’s validity when pancreatic sufficiency is present.

## 4. Resistance and Skepticism Prevail for a Decade

Thus, throughout the 1980s, the CF specialty care field along with geneticists [23] felt generally skeptical about IRT/IRT screening, and many leaders considered CF NBS to be inappropriate or even unethical. In addition to recommendations for more thorough, critical evaluation of IRT, the Cystic Fibrosis Foundation’s Task Force published an emphatic statement in all caps that discouraged CF NBS: “UNTIL SUFFICIENT INFORMATION RELATED TO THESE AND OTHER ISSUES CAN BE OBTAINED, THE TASK FORCE STRONGLY RECOMMENDS THAT NO MASS POPULATION SCREENING FOR CYSTIC FIBROSIS BE IMPLEMENTED, EVEN IF A VALID AND RELIABLE TEST IS AVAILABLE.” This influential committee had a restraining effect on the proliferation phenomenon, but it proved helpful to the Wisconsin team, as we were in the process of organizing and seeking grants for a randomized clinical trial (RCT) to investigate the benefits and risks of an early diagnosis, as well as the test options and their cost. We were convinced that the deep reluctance of CF NBS opponents would not be resolved without convincing evidence of the benefits outweighing the risks that could only come from an RCT.

## 5. The Wisconsin RCT—Design and Planning

### 5.1. Unique Challenges and an Ethical Dilemma

Because there had never been an RCT of any newborn screening initiatives, all aspects of the Wisconsin trial were novel, as we recognized during the intensive design/planning phase of 1983–1984. Challenges such as IRB approvals, parental consent, the avoidance of selection bias, an expedited and complete follow-up, a longitudinal evaluation, and monitoring had to be addressed. An overriding challenge was that the RCT presented legal issues and “A Unique Ethical Dilemma,” [24] particularly with regard to having a control group. These aspects were managed under the leadership of Norman Fost, who supported the practical decisions to employ opt-out consent [24]. Obtaining IRB approvals was time-consuming, since the processes involved countless institutions, including every birthing hospital in Wisconsin, but those efforts led to refinements in the protocol. The most noteworthy enhancement occurred when a clergyman on the IRB of the Medical College of Wisconsin asked if disclosure to parents upon request was acceptable to us, and we agreed immediately to this change as an ethical/legal safeguard. Subsequently, however, one illustrious grant reviewer stated that this provision would “kill the study,” but only 0.03% of parents requested the screening results, and none of them were positive. In fact, the time invested in developing the design and the study operations paid dividends when the RCT was challenged all the way to the U.S. Supreme Court in an unsuccessful case (https://www.casemine.com/judgement/us/59147a20add7b04934404aed; accessed on 24 July 2025). We also benefited from establishing a Data and Safety Monitoring Board (DSMB)—one of the first ever (David DeMets, personal communication).

### 5.2. Hypothesis and Organization

**Figure 1:**
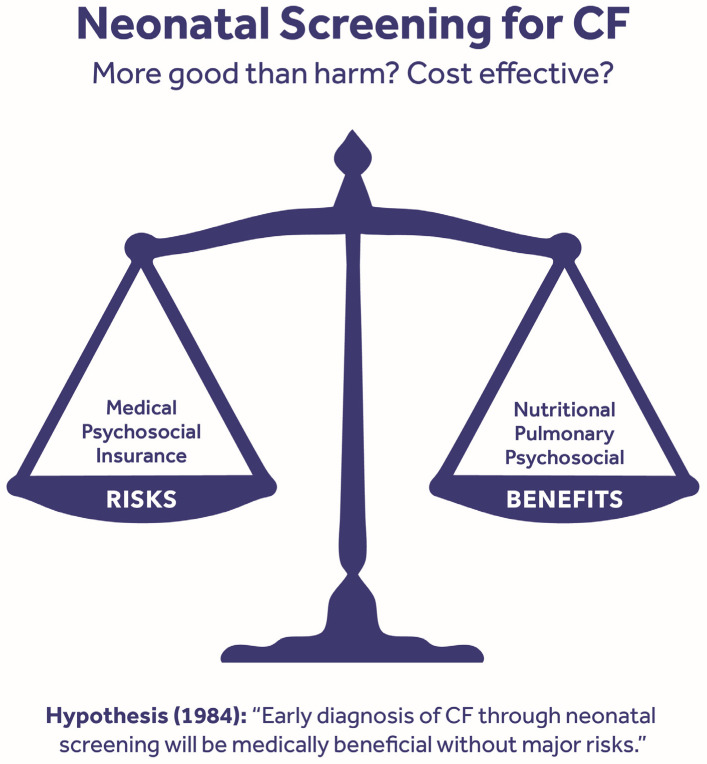
Hypothesis and design of the Wisconsin randomized clinical trial of early diagnoses. Note that the study focused on the benefits and risks of an early diagnosis through newborn screening, but was not intended to be restricted to any single screening algorithm, so transformation to IRT/DNA occurred in 1991.

Fundamentally, the study was designed to determine if an early diagnosis of CF leads to more good than harm and also to estimate its potential cost effectiveness. As shown in Figure 1, the hypothesis was that the “early diagnosis of CF through neonatal screening will be medically beneficial without major risks.” The potential benefits included nutritional, pulmonary, and psychosocial advantages, while the risks studied were medical risks (e.g., the early acquisition of *Pseudomonas aeruginosa*), psychosocial risks, and the potential loss of insurance. Although we attempted to collaborate with two adjacent states, namely Iowa and/or Minnesota, this proved impossible for both laboratory and clinical reasons. Consequently, it was decided to organize a two-institution study—a wise decision—in which the Milwaukee CF Center led by W. Theodore Bruns and the Madison CF Center directed by Elaine Mischler would divide Wisconsin’s birth population into halves for follow-up, and each would use a jointly created evaluation and treatment protocol [25,26] specifying the standard therapies for each enrollee.

For the screening test plan, we benefited greatly from the 2 years of Colorado experience and advice of Keith Hammond, who alerted us about variations and inconsistencies related to the Sorin kit and where to set a fixed cutoff value—a problem because it was set too high [20] and required continuous lowering and, eventually, the floating cutoff calculation method [Overview of Cutoff Determinations and Risk Assessment …—APHL; https://www.aphl.org/programs/newborn_screening/Documents/Cutoff-Determinations-and-Risk-Assessment-Methods.pdf, accessed 26 July 2025).

The randomization scheme was straightforward and effective. When a DBS specimen card arrived at the newborn screening lab and was stamped with a long sample number, the terminal digit determined the group assignment—odd (1, 3, 5, 7, and 9) designated the screened or early-diagnosis group and even numbers were placed in the category we referred to as the control or standard-diagnosis cohort. Note that all the specimens had the screening test performed on the day of arrival, but only the screened group had the result disclosed, while the control data were computer-stored for 4 years unless the parents requested the result or CF was diagnosed based on signs/symptoms or a positive family history. This aspect of the design was critical to avoid selection bias, which invalidated the 5-year “alternate week trial” in the Wales/West Midlands regions [27]; however, our blinding/unblinding design was difficult for some to understand, but clear to the IRBs [24,25].

### 5.3. Outcomes of Interest

It was recognized in the design/planning phase that an evaluation of the nutritional outcomes would be our first and perhaps best opportunity to detect differences within months, and that the nutritional status would be associated with the lung disease risk. But we knew that the variable and uncertain timing of lung disease onset might require many years of evaluation, particularly since spirometry was deferred until 5–7 years of age. Later, we found that spirometry lacked sensitivity because of the FEV1 ceiling effect [25], and this concern led us to develop a longitudinal quantitative chest radiography method [28] that proved invaluable in the years prior to quantitative chest CT examinations.

Complicating the evaluation of lung disease, which had its onset earlier than we anticipated based on both clinical manifestations and imaging data [29,30], it became obvious that numerous intrinsic and extrinsic determinants not related to the age of diagnosis were influencing lung disease onset and progression, as future studies demonstrated clearly [30,31]. Most of these risk factors are listed in Table 1. The most prominent of these was the timing and chronicity of *Pseudomonas aeruginosa* acquisition. To our surprise, the operation of two different kinds of clinics, one of the world’s first segregated clinics that Dr. Mischler organized in Madison and a typical integrated clinic in Milwaukee, provided the rarest of opportunities to discover the serious risk of nosocomial acquisition and the importance of infection control measures. We showed by culture and serology studies that children with CF diagnosed through newborn screening and followed in the typical mixed clinic of the time were at a higher risk for person-to-person pseudomonas transmission [32,33] and that this was detrimental [34]. Recall that the RCT occurred during the era when person-to-person interaction was common with CF camps and other social gatherings, which were strongly recommended until conclusive evidence led to the elimination of those opportunities in 1994.

### 5.4. Accrual of Patients and Observations on Birth Prevalence and IRT Flaws

Randomized screening proceeded beginning on 15 April 1985, and within a few years, our first observation that refuted longstanding dogma became evident—the birth prevalence of CF in a mixed American population is ~1 in 4000, and not the 1 in 2000 claimed for decades [22,35]. During the ensuing decades, it has become obvious that the only reliable method of determining the CF incidence is through NBS. The implication of the 1:4000 finding was clear, as we realized 4–5 years of randomized NBS would not suffice to achieve an adequate statistical power (50 per group), so we eventually needed 9 years to randomly screen 650,341 babies through 30 June 1994.

Our earliest observations also focused on the IRT test and led to the conclusion by 1989 that IRT/IRT is “good but not good enough” [36]. Rock et al. [37] first identified causes of false positive results, such as perinatal stress with low Apgar scores and African American births [37]. Next, Rock et al. [36] in 1990 in a pivotal paper reported age-related IRT declines limiting the time for the second IRT, and most significantly, a disappointing potential for missed cases due to false negative screening results. The same year, because of a variety of concerns, including another ethical dilemma, France suspended their national IRT/IRT program in 1990 under the leadership of Professors Fariaux and Frezel [38]. In fact, there were good reasons at this point to wonder if IRT/IRT had a future in CF NBS.

### 5.5. Benefits of Early Diagnosis

**Figure 2:**
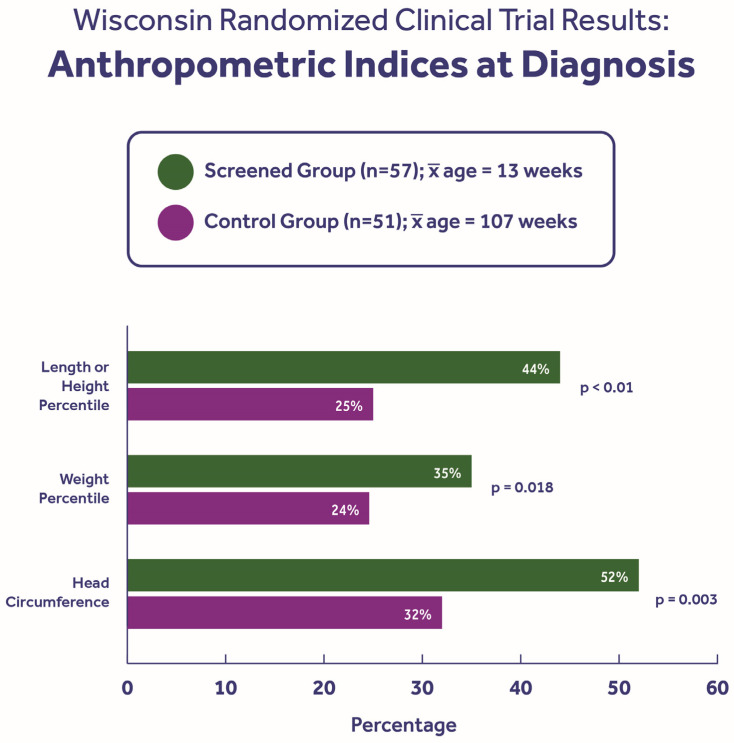
Nutritional benefits of an early diagnosis through newborn screening. The statistically significant differences in height persisted through at least adolescence [43,44], while the head circumference difference was present until two years of age [41].

By March 1994, after nearly 9 years of randomized screening, the nutritional benefits of an early diagnosis were demonstrated [39,40]. As shown in Figure 2, the length/height percentile, weight percentile, and head circumference were all significantly better in the screened group. We were especially impressed by the head circumference difference, which demonstrates that the degree of malnutrition in the cohort that experienced a delayed diagnosis was relatively severe. Moreover, continued evaluation showed that the catchup in head circumference did not occur until 2 years of age [41] and cognitive studies [42] revealed a lower cognitive skill index in the controls that was linked to malnutrition, and particularly to prolonged vitamin E deficiency. Also of great interest was our finding that the stunted growth (lower height) was prolonged through adolescence [43] and potentially into adulthood [44] with a variety of feeding regimens [45].

**Figure 3:**
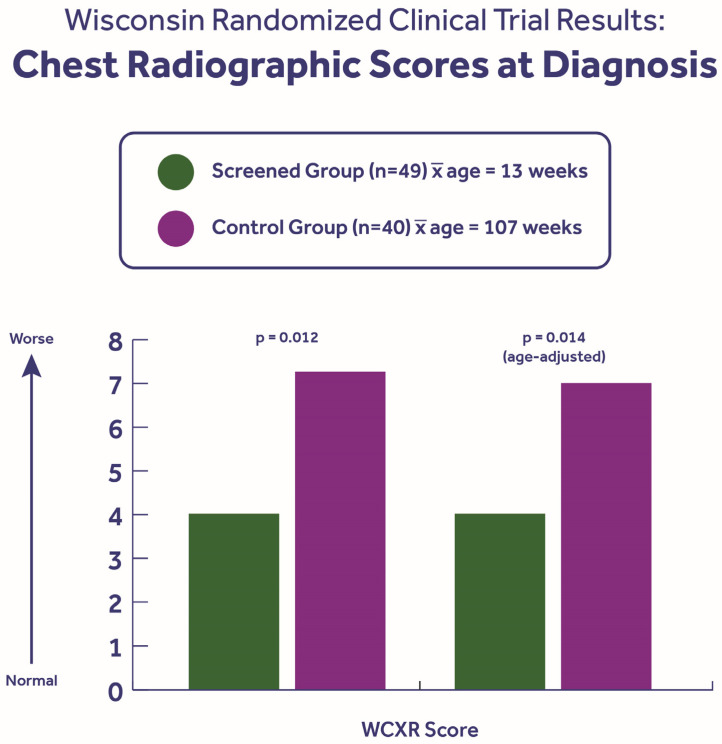
The initial severity of the disease at the time of the diagnosis based on the Wisconsin chest radiograph scoring method [28]. Note that, even with an age adjustment, the screened group showed milder lung disease at the time of the diagnosis, even though they had more severe intrinsic risk based on genotype profiles and the % pancreatic insufficiency [40]. However, with exposure to extrinsic risk factors, especially the differential acquisition of *Pseudomonas aeruginosa* in an integrated clinic, the screened group eventually showed more severe lung disease [30]. There were no gender-related differences.

Figure 3 shows that the screened group also had significantly lower (better) chest radiographic scores at diagnosis. However, as the patients aged, the lung disease severity was similar and then worse in the screened group because of a combination of early pseudomonas acquisition that was associated with bronchiectasis [34], a more severe genotype profile, and a higher percentage of pancreatic insufficiency (91% vs. 73%; *p* = 0.012), despite satisfactory randomization for 16 variables [25,40]. Thus, our study revealed that, although an early diagnosis through NBS can preempt lung disease, i.e., achieve detection before onset, its development and severity are determined by numerous intrinsic and extrinsic factors. These determinants were confirmed in two other studies [30,31] of patients evaluated after NBS and are listed in Table 1. Consequently, Sanders et al. [30] concluded that “*Early diagnosis of CF after NBS provides an opportunity for better care, but does not ensure better outcomes, making NBS necessary but not sufficient to optimize outcomes for the majority of patients with CF*”. Later in a study of 9571 subjects in the CFF Patient Registry who were screened or unscreened, Rosenfeld et al. [46] found that NBS was associated with no lung function differences at age 6 years, in keeping with the above view, but that the screened patients showed a modest improvement in ppFEV1 at 10 years compared to the unscreened group, suggesting either an NBS effect or—more likely, in my judgment—a differential impact of extrinsic determinants over time. That large study confirmed the nutritional advantages of NBS as measured by growth data at 6 and 10 years of age.

In retrospect, it was scientifically fortuitous that two kinds of clinics were employed, and that we were able to demonstrate that segregated clinics are essential to reduce the risk of the person-to-person transmission of respiratory pathogens [25,32,33]. Fortunately, this risk was well recognized by the time other regions began NBS, and the data showed that the risk of pseudomonas acquisition actually seemed lower with an early diagnosis—a phenomenon that has subsequently been linked to breastfeeding [45].

With significant benefits statistically demonstrated, and unlikely to be altered by accruing more control subjects, the DSMB ordered that randomized screening cease on 30 June 1994. Wisconsin then began routine CF NBS on 1 July 1994. More scrutiny of the data and analyses was conducted, and the results were published in *The New England Journal of Medicine* in 1997 [39]. But to complete the RCT, control patient accrual had to continue with continuous “unblinding” and an evaluation of all the control patients until April 1998, after which the definitive statistical analyses were performed. This was followed by the publication of another, strongly confirmatory report [40] that also described the sound randomization scheme.

### 5.6. Risks of CF NBS—Real Potential for Harm

Of the three types of risks that we studied, only the potential psychosocial harm turned out to be a daunting challenge. Our concerns about the loss of medical insurance were eliminated when legislation prevented discrimination based on genetics. Although the risk of acquiring *Pseudomonas aeruginosa* was proven to be significant in the typical integrated clinical environment, our data proved that this could be prevented, and the standard of CF care had already become rigorous with regard to infection control. We were reassured when Rosenfeld et al. [47] reported that there was no increased risk when routine CF NBS programs were evaluated—an observation that was repeatedly confirmed, as reviewed by later by Rosenfeld et al. [46]. Thus, the epidemiologic data we generated on the risks with the two different centers’ organizational care plans were invaluable.

Regarding psychosocial issues, our team was fortunate to have as our first research nurse Audrey Tluczek, who progressed to become an independent investigator and designed/completed/published many studies [48] addressing this potential risk and how to mitigate or prevent harm. Her studies clarified the psychosocial and communication challenges and identified parental preferences and the better methods of communication. Although psychosocial stress cannot be avoided in screening programs, we concluded that the potential harm associated with NBS can be managed with optimized care and communication. In addition, the challenges associated with detecting heterozygote carriers can be effectively managed by genetic counselors.

### 5.7. Wisconsin RCT Impact

The impact of the positive RCT, although not immediate, was substantial. Another state, Massachusetts, began a pilot program with research objectives in 1999, and soon thereafter, New Jersey and New York followed with IRT/DNA protocols. Within a few years, other states began, while European countries, especially the UK, were also directly influenced to proceed. France, which was already poised to resume a national program, proceeded expeditiously and launched an IRT/DNA screening program in 2003 after reorganizing the CF centers that were providing follow-up care. Later, Germany implemented screening. Canada was interesting because Alberta began in 2007 with leadership by Mark Montgomery and British Columbia followed soon thereafter; however, Ontario was resistant [49] and Quebec was delayed for another decade until 2017, when it was accepted that the outcomes were better in the other provinces that were screening [50]—as if the leaders there were unaware of the Wisconsin RCT results and the worldwide implementation of CF NBS.

Surprisingly, however, the CFF remained skeptical, and its leaders did not want to divert attention away from the organization’s care (treatment) and research missions. The leaders’ highest priority at the time was catalyzing and supporting what became CFTR modulator therapy. The CFF was also reluctant to sail into the uncharted waters of the nation’s 50 separate public health programs. Consequently, a decision was made to ask the CDC to weigh the evidence for and against CF NBS—an unprecedented role for this highly respected institution. The first critical workshop [51] occurred in 1997 and led to minimal support for nationwide, universal screening, but a decision was also reached to meet again “in the near future” to reassess the situation. In fact, among other issues, there was great concern that “public health was not ready for genetics” [23,51,52]. But as more states and European countries began CF NBS programs in the first few years of the 21st century, and as CFTR modulator therapy research was showing great promise, the CFF vice president, Preston Campbell, urged the CDC to reconvene for another critical evaluation of the evidence.

The CDC then organized a workshop in November 2003 in which multiple supportive presentations were made that were eventually published [53] after peer review, along with a strong statement of support from the CFF [54]. The CDC report was an MMWR published in October 2004, while the North American CF Conference was being held [53]. The most important statements were as follows:

- *On the basis of a preponderance of evidence, the health benefits to children with CF outweigh the risk of harm and justify screening for CF.*
- *Newborn screening for CF should be accompanied by rigorous infection control practices.*
- *The net balance of benefits and risks is contingent on how newborn screening for CF is implemented.*
- *Newborn screening systems should ensure parental and provider education.*

This and the CFF endorsement had a catalytic effect. Dr. Campbell asked me to become the National Facilitator for Newborn Screening in 2006 and assume responsibility for ensuring that “All states are screening by the end of the decade”—a challenging goal, but one that was accomplished through intensive planning and organization in each state. A strategic decision was made to accept either IRT/DNA, which most states opted for, or the less sensitive, slower IRT/IRT algorithm, which appealed to 12 states that were reluctant to perform DNA analyses and preferred attempting to collect two blood specimens and demonstrate persistently elevated IRT [36]. The eventual transformation of all states to the superior IRT/DNA algorithm nationwide required an additional 15 years, as one by one they recognized the need for DNA analyses for SCID screening [55] and the opportunity to employ the IRT/IRT/DNA strategy developed by Marci Sontag [56], preserving their desire to collect two specimens, despite the risk of delays and losses.

## 6. *CFTR* Gene Discovery and F508del Frequency—Discoveries That Rescued CF NBS

The second pivotal enabling advance in CF NBS occurred when the cystic fibrosis transmembrane regulator gene (*CFTR*) and its principal pathogenic variant, “∆F508” (F508del), was discovered and reported in the September 1989 issue of *Science* [57]. This greatest research achievement in CF history was immediately recognized as providing an opportunity for a better, faster, two-tiered screening test in which the IRT cutoff value could be lowered to improve the sensitivity. In fact, while the second Rock et al. paper [36] was *in press* (then a 6-month process), the editor approved the following commentary to be included under “SPECULATION AND RELEVANCE” as a revision to describing the advantages of the two-tier thyroxin/TSH strategy [15] being used in congenital hypothyroidism NBS:

“*With advances in technology and the recent identification of one of the cystic fibrosis mutations and the identification of other mutations to soon follow, we believe that the strategy for cystic fibrosis newborn screening will need to evolve into a true two-tier screening test. The first tier would be the IRT assay; if the IRT assay is positive, the second tier would be performed on the same original blood spot, and it would be a probe for the cystic fibrosis mutations. The implementation of cystic fibrosis screening, however, should be delayed until a clear benefit of newborn screening has been identified.*”

Our team was expanded quickly by the addition of a superb molecular geneticist, Ron Gregg, and we began the development of the IRT/DNA (F508 del) method [58,59] while concurrently showing that nearly 90% of Wisconsin’s 547 patients carried this variant. Initially, we used the rapid polyacrylamide gel technique reported by Rommens et al. [60] after the extraction and PCR steps, but we eventually changed to a probe methodology. Concurrently, Enzo Ranieri in Adelaide, Australia, developed a similar method [61], and soon thereafter, Claude Férec’s team in Brest developed a method as well [62]. The two-tiered strategy allowed programs to lower the cutoff value for IRT to prevent some of the false negative results.

### 6.1. Expansion of DNA/CFTR Panels

A DNA analysis for only the F508del variant was used for about the first decade of IRT/DNA screening, but the initial two-tiered method limited the sensitivity to about 90% at best. The next important advance was the widespread implementation of the *CFTR* multi-mutation method [63], which eventually included the 23 variants recommended by the American College of Medical Genetics in a revised statement of 2004 [64] correcting an earlier 25-variant recommendation. Easy-to-use kits became available and countries like France employed *CFTR* panels that matched their CF population genetics [65]—an important concept to employ. Thus, the evolution of DNA/*CFTR* from solely F508del-variant detection to expanded panels that attempted to cover regional CF patient populations became the standard of NBS practice, even though concerns were raised about the detection of more *CFTR* variant carriers, especially in Europe. In addition, cautions are necessary regarding labs inaccurately identifying variants, and thus, the confirmation of genotype is ideal with venipuncture-obtained blood as part of the CF diagnostic process when a sweat test is performed.

### 6.2. CFTR Gene Sequencing to Expand Panels Further

For the next decade, most programs in the USA used a kit with 23 pathogenic variants. An exception was in California [66], where a novel methodology was introduced in which a panel of 40 initial variants, and ultimately, 77, was used in the second tier when the IRT concentration exceeded a value set at the 98th percentile. Next, whenever a single variant was found, *CFTR* Sanger sequencing was performed and only babies with two variants were designated as positive. The sequencing/reporting was performed for any variant, whether known to be pathogenic or not. This led to a maximum sensitivity of 92%, which was limited by the size of initial *CFTR* panel, and a high proportion of cases with cystic fibrosis transmembrane conductance regulator-related metabolic syndrome (CRMS) [67], which was later designated as cystic fibrosis screen positive, inconclusive diagnosis (CFSPID) [68]. Other CF NBS algorithms have also led to many more CRMS/CFSPID cases than CF [69], as this condition has puzzled programs about the best way to manage these diagnostic dilemmas, despite the published guidelines [68,69]. Indeed, one can argue that CRMS/CFSPID has become a distraction away from more QI to address the challenges of equity, timeliness, and optimized infant care, and that this enigmatic condition is contributing more harm than good, particularly in the widely varying ways that it is being managed medically.

### 6.3. The CFTR2 Project’s Important Role in NBS

In view of the California experience and growing knowledge about CF diversity, an important contribution to CF in general and CF NBS in particular has been the project entitled The Clinical and Functional TRanslation of *CFTR* or CFTR2 (CFTR2, http://www.cftr2.org/—accessed on 30 June 2025). This timely, invaluable initiative at Johns Hopkins University advanced the initial *CFTR* variant repository website maintained at Toronto’s Hospital for Sick Children (Cystic Fibrosis Mutation Database, http://www.genet.sickkids.on.ca/cftr/app—accessed on 30 June 2025) by determining the pathogenicity of variants in Garry Cuttings’ program with operational leadership by Karen Raraigh. As reported by Sosnay et al. [70], this invaluable resource has classified variants for the past 15 years into categories of CF-causing mutations, variants with varying clinical consequences (VVCCs), and, until recently, variants of unknown significance (VUSs). The CFTR2 project reported on 22 July 2013 that 159 *CFTR* variants account for 96.4% of CF alleles [70]. As of 25 September 2024, the CFTR2 resource includes data from 122,935 patients representing 55 regions and covers all ethnicities well except for Asians while annotating 1167 variants, 1085 of which are CF-causing and 55 of which are VVCCs. The VUS category has been eliminated. A similar program in France [71] has added to the knowledge of *CFTR* variants, and together, these resources have become increasingly important with the advent of next-generation sequencing. The Hopkins and French programs, which have provided reliable *CFTR* variant classification, are already providing an invaluable asset and will become even more important in the future with the advent and growing use of next-generation sequencing.

### 6.4. Next-Generation Sequencing

When the commonly used Hologic 23 mutation kit was abruptly withdrawn on 31 March 2015, larger panels with 39 or 60 variants were introduced in the USA by Luminex. Some European countries like France were already using larger *CFTR* panels available in Elucigene kits. In 2014, Baker et al. [72] determined that next-generation sequencing (NGS) using a sound FDA-approved Illumina method could be applied for routine complete *CFTR* gene sequencing analyses in the Wisconsin State Laboratory of Hygiene with a 139–162-variant panel. Soon thereafter, the size of the panel was readily increased with no additional costs by modifying the reporting software and the selection of variants, refined to avoid VVCCs and VUSs and reduce the detection of CRMS/CFSPID cases [73]. Experience over a decade in Wisconsin has shown that the IRT/NGS algorithm is affordable and can be accomplished with a satisfactory turnaround time [73]. New York has also used NGS in a third-tier strategy but detects VVCCs and identifies three CRMS/CFSPID cases for each CF patient diagnosed [74].

**Figure 4:**
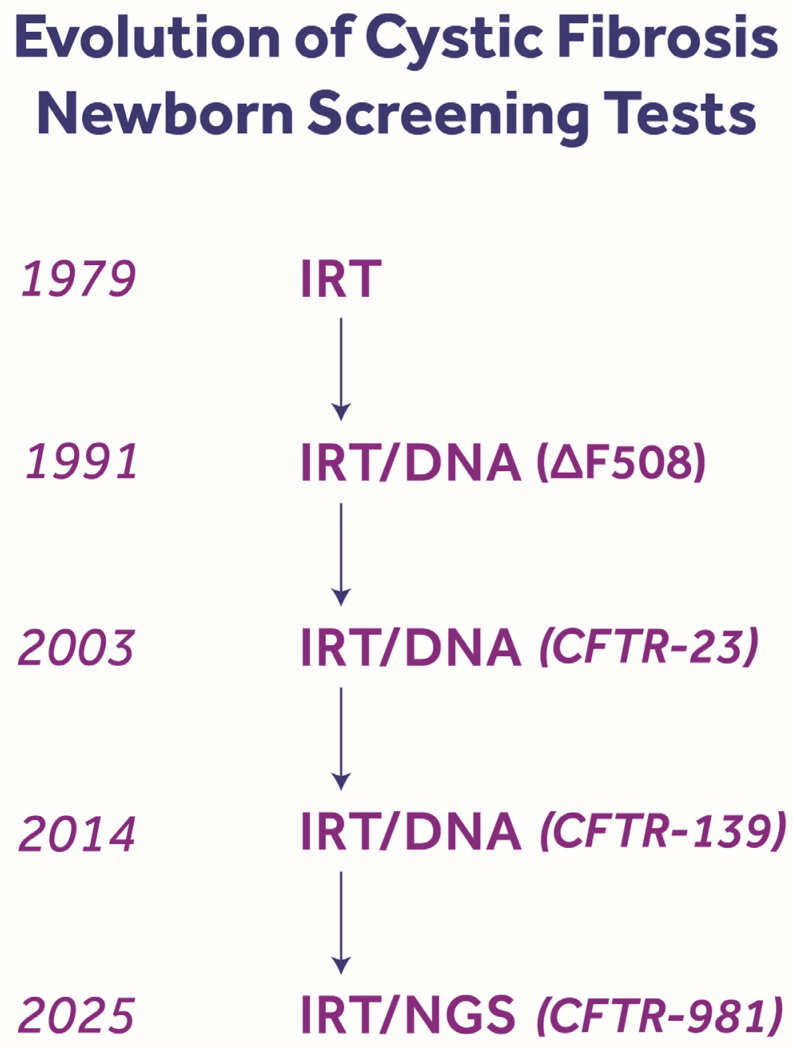
Evolution of cystic fibrosis newborn screening tests, using dried blood spot specimens. Note that the IRT/IRT method was used during the 1980s until it was supplanted by IRT/DNA, with the F508del allele being the only *CFTR* variant identified in the second tier. The 2-tier method has remained the most used algorithm for the past three decades as the *CFTR* panel has been continually expanding with a variety of kits followed by the introduction of next-generation sequencing in 2014 to detect 139 variants originally [72], and during 2025–26, as many as 981 pathogenic variants [73]. Some screening laboratories add an additional biomarker step to IRT/DNA as a “failsafe” measure.

Thus, the CF NBS algorithm has progressed dramatically during the past 45 years, as shown in Figure 4. During 2025 Wisconsin increased the *CFTR* panel from 689 to 981 CF-causing variants listed on the CFTR2 website while New York increased their panel to 1018 *CFTR* variants. But using NGS technology requires special expertise, as exemplified by Mei Baker in Wisconsin and Denise Kay in New York where CFF-sponsored Cystic Fibrosis Newborn Screening Genetic Testing Resources Centers have been established. In addition, not all NBS labs have the volume of specimens needed to analyze in an efficient, cost-effective manner, so some programs will outsource NGS (e.g., Kansas sends high-IRT specimens to Wisconsin for NGS). The evolution of NGS with better operational technologies can be expected in the future to improve the analytical time, accuracy, and applicability.

## 7. Equity and Timeliness

The long-standing dogma that CF is a “white person’s disease” was soundly refuted by a research team assembled by Susanna McColley [75], who studied 6354 children diagnosed through NBS from 2010–2018 and reported to the CFF Patient Registry. This revealed that about 20% of the cohort were African American (~7%) or Hispanic (~13%) babies [76]. Despite NBS, the diagnoses were later in the minoritized populations, and they suffered more disparities, including malnutrition [75,77].

In general, however, timeliness has not been given sufficient priority, and many diagnoses are made after the 28-day neonatal period, whereas other genetic disorders on the screening panel are generally diagnosed by 1–2 weeks of age with more efficient follow-up programs than CF centers have achieved. There are many reasons why CF NBS programs have experienced and tolerated delays. In fact, the ECFS standards [78] state that “The majority of infants with a confirmed diagnosis after NBS should be seen by the CF specialist team by 35 days and no later than 58 days after birth.” Longer delays are attributable to detecting babies with a single mutation, geographic factors, weather, sweat testing limitations, and two sample testing in the IRT/IRT/DNA algorithm or other multistep methods [56,79]. Although it is more difficult to obtain sweat at 2 weeks of age than a few weeks later, most sweat tests performed then should be successful. In addition, with expanded *CFTR* panels identifying CF cases with two pathogenic mutations, a presumptive genetic diagnosis can be made and treatment initiated rather than being delayed, as McColley [75] has emphasized.

## 8. Current Situation

### 8.1. Successes to Appreciate and Celebrate

After 45 years of using IRT/IRT or IRT/DNA, there is much to celebrate because of numerous successes, as listed in Table 2. Clearly, an earlier diagnosis of most CF infants with concurrent genotyping is an extraordinary success. A dramatic example in the USA was described to me recently—a baby born in Georgia was seen at the Emory CF clinic and diagnosed genetically at 8 days with a F508del/G551D genotype, and then, after pre-therapy testing, was placed at 5 weeks of age on ivacaftor, the first CFTR modulator drug approved for infants in May 2023 (Rachel Linnemann, personal communication). This baby benefitted dramatically from the doubly transforming impact [80] of NBS and CFTR modulator therapy and illustrates what will be ideal for all infants with CF in the future.

Routine genetic counseling has been another success story [81,82]. Clearly, the quality of life of children with CF has been enhanced, especially since fewer hospitalizations are needed. Other advances include avoiding the stressful “diagnostic odyssey” [53] and creating the opportunity for better nutrition early and the attainment of genetic growth potential as the patients age [83], which was demonstrated as a benefit of NBS in the Wisconsin RCT [43,44,84]. An early diagnosis will not prevent lung disease with its many risk factors [30,31], but it will provide an opportunity for preemptive therapy and an ability to lower the potential for hospitalizations while improving the quality of life. We should also celebrate the fact that optimism among parents and children is now routine. Lastly, on a system level, important advances include strong partnerships with NBS labs, the better organization of care centers, and an increased understanding of the disease.

### 8.2. Shortcomings—Needs/Opportunities

Although the CF NBS field can rightly be proud of the many achievements, there are shortcomings in the screening tests employed and outcomes that should be addressed, as listed in Table 2. The needs and opportunities include overcoming the long-standing problem of false negative results due to IRT and inadequate *CFTR* panels [84,85]—a failure that does not plague NBS for other genetic conditions. But CF NBS programs have always tolerated a relatively low sensitivity by other screening standards rather than striving for 100% sensitivity. Achieving equity remains crucial, especially for non-White babies with CF and for neonates served with less-than-optimal screening algorithms as described in the new CFF guidelines recommending improvements [84]. Timeliness should require a diagnosis by 1–2 weeks of age, as with other genetic conditions, so that special nutritional needs can be met quickly. Because evidence of malnutrition is present in most 2-week-old babies with CF and pancreatic insufficiency, and in view of the risks of dehydration with salt loss in hot climates, timeliness through more efficient follow-up should be given higher priority.

Despite decades of research, infant nutrition practices still need to be improved after an early diagnosis. Recent data from the FIRST project led by HuiChuan Lai confirming the pulmonary advantages of breastfeeding [45,86] should alert the field that every baby with CF deserves to be breastfed. Improvements are needed in sweat testing and the judicious selection of medications. There is also a need to extend CF NBS globally in low- and middle-income countries where it will be worthwhile [79]. Lastly, there needs to be a reduction in CRMS/CFSPID cases so that programs can better focus their QI efforts on addressing the equity, timeliness, and optimized infant care while reducing the harm of CF NBS.

## 9. Predictions Without a Timeframe

This analysis examined critically when and why CF NBS started, where we are now, how we got here, and what more is needed, and it should allow for predictions about what is on the horizon as quality improvement continues. But screening labs are slow to change, and CF clinicians are often conservative, so it would be hazardous to forecast within a timeframe. Consequently, I wish to share my personal/professional views on predicted advances without a timeframe. The five predictions described below are intended particularly for the USA but may also apply to some progressive European countries like Norway and the Netherlands.

- CF NBS will evolve into an equitable, more sensitive, and specific NGS-based primary DNA (genetic) test if the ethical, legal, and social issues of detecting *CFTR* variant carriers are resolved and public acceptance occurs—both of which are likely, in my opinion. This prediction was actually made by my CDC mentor, Harry Hannon, as analytical molecular biology techniques made whole-genome sequencing feasible. I believe that some NBS labs will bundle variants for a variety of genetic disorders, as some programs are now doing on a research basis [87,88], and that this will become increasingly desirable and affordable for NBS labs. The inadvertent detection of some disorders with no therapy currently available as well as single variant cases of diseases like CF can be managed by informatics filters, as we have done in whole-genome sequencing studies [31]. And carrier detection may become desirable if/when it is accepted that carriers have disease risks, as has become true for *CFTR* variant carriers [89]. But NGS will need to be augmented by methods to detect genomic structural abnormalities like duplications and deletions as well as significant intronic variants.
- CRMS/CFSPID will become much less significant if the *CFTR* panels are “refined,” as Rock et al. [73] recommended, or if IRT is no longer used an analyte. In fact, IRT is a flawed biomarker [85,90,91] and may eventually be abandoned. If the lessons of history apply, IRT will indeed be supplanted by better technology like NGS.
- CF diagnoses will be made routinely (genetically) by 1–2 weeks of age based on identifying two pathogenic variants, thus facilitating earlier/better care, including breastfeeding [45,86,92] and the early administration of nutritional supplements to avoid malnutrition and its consequences, as well as the initiation of infant-approved CFTR modulator drugs like ivacaftor. There will be many advantages of beginning care by 1–2 weeks of age, when babies are more likely to be breastfed successfully with support by CF centers and primary care physicians [86,92].
- Improved therapy for infants will occur through better CFTR defect management drugs, as they are being improved continuously and will become more affordable with potential injectable administration to overcome non-adherence. Although drugs like ivacaftor and trikafta can be very effective, not all patients benefit, and gene editing may become more appealing.
- After the diagnosis and initiation of treatment, the increasingly routine care of many, if not most, children with CF will eventually be conducted predominantly by pediatricians in association with primary care delivery [80] and in close collaboration with CF center specialists for decisions about highly specialized therapies like CFTR-directed drugs. Pediatric pulmonologists are likely to be needed less, with lung disease being prevented, but a role for CF subspecialists in confirming diagnoses and managing complex interventions will remain.

## Figures and Tables

**Table 1:** Potential risk factors for onset and severity of CF lung disease.

**Intrinsic Determinants**
• Genotype
• Modifier genes
• Pancreatic status
• Meconium ileus
• Nutritional status
**Extrinsic Determinants**
• Age in weeks at diagnosis
• Environmental exposures
• Parental education, especially the level of maternal education
• Socioeconomic status
• Daycare
• Integrated clinic
• *Pseudomonas aeruginosa*
• *Staphylococcus aureus*
• Height percentile < 10th
• Hospitalizations
• Pulmonary exacerbations

**Table 2:** Current status of CF newborn screening programs.

**Accomplishments**
• Earlier diagnoses routinely for most patients
• Avoiding the “diagnostic odyssey”
• Concurrent genotyping with IRT/DNA
• Prompt access to CF specialist care
• Early GI/nutrition Rx (PERT and nutrient supplements)
• Preempting lung disease
• CFTR modulator therapy for infants (e.g., ivacaftor)
• Opportunity for genetic counseling
• Fewer hospitalizations
• Much improved quality of life
• Optimism among parents/children
• Partnerships of CF specialists with NBS lab leaders
• Better organization of care centers
• Increased understanding of the disease
**Needs/Opportunities**
• Overcome 5–20% false negative NBS results
• Reduce false positive results
• Achieve equity everywhere (especially eliminate racial/ethnic disparities)
• Improve timeliness (diagnose by 1–2 w/o)
• Improve nutrition-related outcomes through breastfeeding and supplements
• Reduce sweat-testing failures
• Avoid excessive medications
• Overcome variations in follow-up efficiency and geo-barriers
• Extend globally where worthwhile
• Reduce CRMS/CFSPID
